# Correction: Coating effect of renatured triple-helix lentinan on the morphology and antimicrobial activity of ZnO synthesized by hydrothermal method

**DOI:** 10.1039/d4ra90080d

**Published:** 2024-07-24

**Authors:** Xuewei Jia, Yihong Wu, Zhiyang Liu, Yuxiang Dai, Tianxiao Li, Mingqi Gao, Chunping Xu

**Affiliations:** a Zhengzhou University of Light Industry Zhengzhou Henan China c.p.xu@zzuli.edu.cn +86 371 86609637; b China Tobacco Henan Industrial Co., Ltd Zhengzhou Henan China gaomq1984@126.com +86 371 61291027

## Abstract

Correction for ‘Coating effect of renatured triple-helix lentinan on the morphology and antimicrobial activity of ZnO synthesized by hydrothermal method’ by Xuewei Jia *et al.*, *RSC Adv.*, 2024, **14**, 17814–17823, https://doi.org/10.1039/D4RA01590H.

The authors regret that the affiliation of one of the authors (Zhiyang Liu) was shown incorrectly in the original article. The corrected author list and affiliations are as shown here.

The Royal Society of Chemistry apologises for these errors and any consequent inconvenience to authors and readers.

